# Lifestyle Factors in Myopic Spanish Children

**DOI:** 10.3390/children11020139

**Published:** 2024-01-23

**Authors:** Noemí Güemes-Villahoz, Rosario Gómez de Liano, Paloma Porras Ángel, Paula Talavero González, Rafael Bella Gala, Beatriz Martín García, Bárbara Burgos Blasco, Elena Hernández García, Marta Chamorro Herrera, José Luis Hernández-Verdejo, Alicia Ruiz-Pomeda

**Affiliations:** 1 Department of Ophthalmology, Instituto de Investigación Sanitaria del Hospital Clínico San Carlos (IdiSCC), C/Profesor Martin Lagos S/N, 28040 Madrid, Spain; noemi.guemes@salud.madrid.org (N.G.-V.); dra.rosario@gomezdeliano.com (R.G.d.L.); palomapo@ucm.es (P.P.Á.); paula.talavero@salud.madrid.org (P.T.G.); ehernandezg@salud.madrid.org (E.H.G.); martacha@ucm.es (M.C.H.); 2 Optometry and Vision Department, Faculty of Optics and Optometry, Complutense University of Madrid, 28037 Madrid, Spain; rbella@ucm.es (R.B.G.); bmarting@ucm.es (B.M.G.); jlhernan@ucm.es (J.L.H.-V.)

**Keywords:** lifestyle factors, myopia, children, screen time, near work, outdoors

## Abstract

Background: Childhood myopia represents a global concern with increasing prevalence in recent decades. Lifestyle factors significantly impact myopia. Aim: To evaluate lifestyle factors in myopic children from a metropolitan area in Europe. Methods: This was a descriptive study including myopic subjects aged 4–18 years. Patient demographic and clinical data were collected, including cycloplegic refraction in spherical equivalent refraction (SER) and axial length (AL). In addition, a questionnaire on lifestyle factors was conducted between September 2022 and April 2023. Results: A total of 321 myopic children were included, aged 10.72 ± 3.05 years, of whom 51.4% were boys, with SER −2.25 ± 1.9 D and AL 24.54 ± 0.98 mm. The mean age of myopia onset was 7.69 ± 3.05 years. A total of 59.8% had family history of myopia. Those children who had <2 h/day of screen time (on weekdays) presented SER −2 ± 1.91 D, compared to those who had >2 h/day, SER: −2.50 ±1.88 D (*p* = 0.009). Children who spent <2 h/day doing near work after school were less myopic compared to those who spent >2 h/day (SER: −1.75 ± 1.83 vs. SER: −2.75 ± 1.82, respectively, *p* = 0.03). However, no significant association was observed between SER and AL and time spent outdoors nor between SER and AL and academic performance (*p* > 0.05). Conclusions: Screen time and near-work time appear to be lifestyle factors related to myopia.

## 1. Introduction

Myopia is one of the most common ocular disorders worldwide [1,2,3], showing increasing prevalence in the last decades [4]. An excessive elongation of the eyeball is associated with an increased risk of pathological ocular changes such as myopic maculopathy and retinal detachment, which can lead to significant visual loss vision and blindness, especially in cases of high myopia [5].

To provide a framework for research into myopia prevention, the International Myopia Institute (IMI) has defined “Low myopia” as “a condition in which the spherical equivalent objective refractive error is ≤−0.50 D in either eye” and “High Myopia” as “a condition in which the spherical equivalent objective refractive error is ≤−6.00 D in either eye” [6]. High myopia is also diagnosed when axial length (AL) is equal to or greater than 26 mm, which is usually associated with pathological axial elongation and progressive chorioretinal degeneration of the posterior pole [7].

The prevalence of myopia has increased considerably in recent decades. According to a systematic review and meta-analysis, in 2010, myopia affected 28% (1950 million) of the world population, in 2020, 34% of the population, while the most recent projection estimates that 50% (almost 5 billion) will be myopic by 2050 [4,8]. As for European data, myopia is becoming more common; a meta-analysis of cross-sectional population-based studies from the European Eye Epidemiology Consortium reported an increase in the prevalence of myopia in more recent birth decades; the prevalence of age-standardized myopia increased from 17.8% in those born between 1910 and 1939 to 23.5% in those born between 1940 and 1979 [9]. This progressive increase has led to myopia being considered a public health problem. The expected increase in the magnitude and number of cases of myopia is believed to generate economic and social repercussions due to its high incidence and progressive trend [8,10].

The development and progression of myopia involve a complex interplay of genetic and environmental factors, many of which do not exert the same influence on its onset and worsening [11,12,13,14,15,16]. Parental myopia is a known risk factor for childhood myopia development, suggesting a genetic contribution [5]. However, the increased prevalence of myopia appears to be primarily influenced by environmental and lifestyle factors, including reduced outdoor activities (linked to decreased sunlight exposure), increased near-work activities, and working in poor light conditions [11,14,17]. A recent systematic review reported that lifestyle factors, including increased near work, less outdoor activity, and attending schools with intensive schedules were found to be risk factors for myopia [18]. Activities that involve spending more time (>30 min) on near work, such as reading, studying, or using electronic devices, are associated with an increased risk of myopia [19]. The World Health Organization (WHO) has recognized gaming disorders as a disease in the 11th revision of the International Classification of Diseases-11, and the potential impact of excessive screen time on the development of myopia, especially in school-age population, possibly combined with a lack of outdoor exposure, is a matter of concern [11]. Meanwhile, increased outdoor activity (>40 min/day), especially during daylight hours, has been shown to have a protective effect against myopia [19]. In Spain, this increasing trend has also been described in several population-based studies [20]. Alvarez-Peregrina et al. [21] investigated the lifestyles of 6152 Spanish children, reporting that 43.3% of the participants spent more than 3 h a day doing near-work activities, and 48.9% of this group spent more than 50% of this time using electronic devices. Only 9.7% spent more than 2.5 h outdoors each day. In a cohort of Spanish university graduates, the mean time of exposure to computers was 14 h/week and this exposure to computer use was associated with myopia development or progression [22]. However, the lifestyles of myopic Spanish children has not been thoroughly investigated nor has the correlation of lifestyle with myopia characteristics.

The main objective of this study was to evaluate the lifestyles of myopic children in a metropolitan area of a European capital, i.e., Madrid, Spain. For this purpose, lifestyle habits (near vision work, outdoors time, school performance, etc.) and their relationship with myopic refractive error (age of onset, magnitude, progression, history, etc.) and axial length were evaluated. Considering that lifestyle factors may be modified or adjusted, it is particularly important to better understand their role in the onset and progression of myopia.

## 2. Materials and Methods

This cross-sectional study was conducted in myopic children at the Pediatric Ophthalmology Department of Hospital Clinico San Carlos, Madrid, Spain. This study was approved by the hospital’s Clinical Research Ethics Committee and was conducted in accordance with the Declaration of Helsinki. Written informed consent was obtained from all parents and/or guardians and informed assent from all pediatric patients. This study is a pilot study based on the clinical trial protocol “EUCTR2021-003373-64-ES” approved by the Ethics Committee of Hospital Clinico San Carlos, Madrid, Spain (protocol code EUCTR2021-003373-64-ES and date of approval 1 September 2021). Informed consent was obtained from all subjects involved in this study.

Inclusion criteria were as follows: age 4–18 years; cycloplegic autorefraction with a myopic error equal to or greater than SER: −0.50 D, less than −2.00 D of astigmatism, and less than 1.50 D anisometropia; corrected distance visual acuity (CDVA) of 0.18 LogMar (6/9 Snellen notation in meters) or better; signed informed consent by parents or guardians in all patients and by patients between 12 and 18 years old as well as assent in children under 12 years of age. Children with manifest strabismus, binocular vision anomalies, ocular disorders of the anterior or posterior segment that affected CDVA, amblyopia, previous ocular surgery, or systemic pathologies (cardiopulmonary disease, connective tissue disorders, neurological, or psychiatric disorders) were excluded.

All the children who participated in this study underwent a complete ophthalmologic examination and completed a questionnaire from September 2022 to April 2023 to assess potential environmental risk factors for myopia.

The questionnaire included questions about age, sex, personal history (prematurity, medical and ophthalmological history, child’s growth and development, iris color, age of onset of myopia, previous ocular surgery, previous treatment of myopia, school performance), family history of myopia and lifestyle factors related to myopia, such as number of hours outdoors per day during weekdays and weekends, use of electronic devices at school, school performance (outstanding, adequate, and mild and severe difficulty), school schedule (split, intensive, or mixed), extracurricular activities (outdoors, indoors, both), average screen time (weekdays and weekends), near-work time (after school), lighting conditions during near work at home (natural light, ceiling illumination, desk lamp), and access to green spaces while at home. The questionnaire was conducted by the same experienced researchers (N.G.-V., A.R.P., P.P.A., P.T.G.) and answered by the parents with the children being present. The ophthalmologic exam consisted of the following tests: monocular and binocular visual acuity (VA) with and without best corrected refraction using the ETDRS and LogMar scales, subjective refraction (distance and near), objective cycloplegic refraction in spherical equivalent refraction (SER) measured with an autorefractometer (Huvitz autoref/keratometer HRK-7000A Huvitz Co., Ltd., Gunpo, Republic of Korea), and axial length (AL) measured with an optical biometer (Lenstar LS 900, Haag-Streit, Koeniz, Switzerland). Simple myopia was defined as an SER less negative than −6.00 D, while high myopia was considered as a SER equal to or more negative than −6.00 D and/or axial length (AL) ≥ 26 mm.

In addition, distance and near cover tests was performed with a prism bar, and near point of convergence and stereopsis were evaluated, the latter using the TNO stereo test wearing red–green glasses. Fusion was evaluated with the Worth 4-Dot test. Slit-lamp examination was performed to observe the anterior segment and the fundus, and intraocular pressure (IOP) was measured with Icare ic100 rebound tonometer (iCare Finland Oy, Helsinki, Finland). Cycloplegic refraction was performed after the cycloplegia regimen, which consisted of 3 drops of 1% cyclopentolate separated by 5 min. Cycloplegic refraction was obtained 30 min after the third drop. If necessary, further cycles of cycloplegic eye drops were administered to ensure proper pupil dilation. The cycloplegic refraction obtained was expressed in terms of spherical equivalent refraction (SER). Finally, fundus examination and optical coherence tomography (OCT) of the optic nerve and the macula were performed (Spectralis OCT, Heidelberg Engineering, Heidelberg, Germany).

Statistical analysis was performed using the software package SPSS^®^ (Statistical Package for Social Sciences, v25.0; SPSS Inc., Chicago, IL, USA). Qualitative variables were presented using frequency distribution, while quantitative variables were depicted using means and standard deviations. To study the lifestyle factors associated with myopia, a chi-square test was used for qualitative variables. To study the association between qualitative variables (2 groups) and quantitative variables, Student’s *t*-test or the Mann–Whitney test were used depending on whether the variables were normally distributed. To investigate correlations, Pearson’s rho or Spearman’s rho were used depending on the distribution of the variables. Statistical significance was set at 0.05. Sample size estimation was performed with the “GRANMO sample size calculator”. It was based on data from previous studies with European [23,24,25] and Spanish myopic children [20]. Taking a statistical power of 0.95 and assuming a standard deviation of the axial length of 0.9 mm, a sample size of 312 subjects was needed. Finally, it was decided to recruit up to 321 children in case there were any subjects with incomplete data.

## 3. Results

### 3.1. Demographic Data

A total of 321 consecutive myopic children who agreed to participate in this study and met the inclusion criteria were recruited at the Pediatric Ophthalmology Service of Hospital Clinico San Carlos in Madrid, Spain, between September 2022 and April 2023. Overall, the average age was 10.72 ± 3.17 years, and 51.4% were boys. The mean age of myopia onset was 7.69 ± 3.05 years, and the age of onset of myopia was less than 10 years in 69.7% of the children in this study. Data from the right eye of each patient were used unless the data from the right eye did not meet the inclusion criteria or were not available, in which case, the data from the left eye were analyzed. Mean cycloplegic refraction (SER) was −2.25 ± 1.90 D (min: −0.50; max: −11.7 D) and axial length was 24.54 ± 0.98 mm (min: 22.12; max: 27.85). Ninety-two percent of all the children had simple myopia and eight percent had high myopia and non-pathological myopia. Differences between subjective dry refraction and automated cycloplegic refraction were statistically significant (*p* < 0.001 for both right and left eyes).

Table 1 depicts the personal and family histories of the children included in this study and Table 2 shows the lifestyle factors related to myopia.

#### Homogeneity Analysis

Homogeneity analysis with respect to sex and age variables shows equal groups in relation to cycloplegic refraction (*p* > 0.05), and statistically significant differences (*p* < 0.001) are shown in relation to axial length, which was more frequent in the male sex (AL: 24.76 ± 0.98 mm in boys vs. AL: 24.29 ± 0.93 mm in girls), for which reason an adjusted analysis was carried out for this variable.

Axial length was correlated with myopia using Pearson’s coefficient, yielding a Spearman’s rho of −0.624 for the right eye and −0.504 for the left eye. Thus, there was a moderate inverse correlation between the two variables, showing that axial length moderately increased with higher myopia.

### 3.2. Association between Myopia and Axial Length Rates with Lifestyle Factors

Table 3 shows the relationship of the medical and ocular history variables and lifestyle factors with cycloplegic refraction and axial length in the analyzed subjects. To account for possible confounding factors, the results were adjusted for age and sex where necessary with a linear regression analysis.

There were no statistically significant differences in the following variables: medical history, prematurity, growth retardation, parental myopia, previous eye surgery, current medical treatment, and ophthalmological history (*p* < 0.05).

As for parental myopia, three groups were considered: those in whom neither parent had myopia, those in whom either the mother or the father were myopic, and those whose parents were both myopic. There were no differences in cycloplegic refraction and axial length between these three groups.

Regarding the age of onset of myopia, children who began to be myopic before the age of 10 had more myopia (SER: −2.5 ± 1.99 D) than children whose age of onset was after 10 years (SER: −1.75 ± 1.44 D) (*p* < 0.001), while there were no significant differences in terms of AL (*p* = 0.667). Children who had undergone any prior myopia treatment had more myopia and their axial length was greater (SER: −3.0 ± 2.33 D/24.84 ± 1.01 mm) than those who had not undergone treatment (SE: −1.75 ± 1.62 D/24.43 ± 0.96 mm) (*p* < 0.0001 and *p* = 0.002, respectively).

In addition, the questionnaire on school performance, school schedule, use or non-use of devices at school, and extracurricular activities was also included and the answers were correlated with myopia and axial length. The questionnaire asked about the average school performance of the child according to three categories: score from 9 to 10: outstanding; score from 5 to 8.9: adequate; and score from 0 to 4.9: mild or severe difficulties. Three groups were established to investigate whether there was any correlation between school performance and myopia magnitude and axial length: outstanding (39.3%), adequate (47.6%), and, finally, the group with mild or severe difficulty (12.5%). Descriptive analysis found that the group with mild or severe difficulty had greater axial length than the other two groups, but in subsequent statistical analysis of these data, no significant differences were found between the groups (*p* > 0.05).

The type of school schedule was also evaluated. School hours in Spain are generally divided into three types of programs: a continuous school day (classes approximately from 8 a.m. to 2 p.m.), a split school day (from 9 a.m. to 1 p.m. and then from 3 p.m. to 5 p.m.), and a mixed school day, which is a combination of the two mentioned above. Our study found that 51.3% had a split schedule, 46% had a continuous schedule, and 2.3% had a mixed schedule. The results showed that there were no significant differences depending on school hours (*p* > 0.05).

Of the total number of participants included, 56.3% did not use electronic devices at school, while 43.7% did (Table 2). The group that did not use devices at school had SER −2.0 ± 1.77 D and AL 24.46 ± 1.01 mm, and the group that did use them had SER −2.50 ± 2.05 D and AL 24.63 ± 0.96 mm, *p* = 0.071 and *p* = 0.135, respectively. These results are therefore not significant, although at the descriptive level, it seems that there may be a relationship (Table 3). In relation to extracurricular activities, 26.1% did not engage in any, 20.5% engaged in outdoor activities, 38.6% took part in activities in indoor spaces, and 14% attended activities both indoors and outdoors. There were no significant differences in terms of refraction or axial length in relation to whether or not the children participated in extracurricular activities (*p* > 0.05).

**Figure 1:**
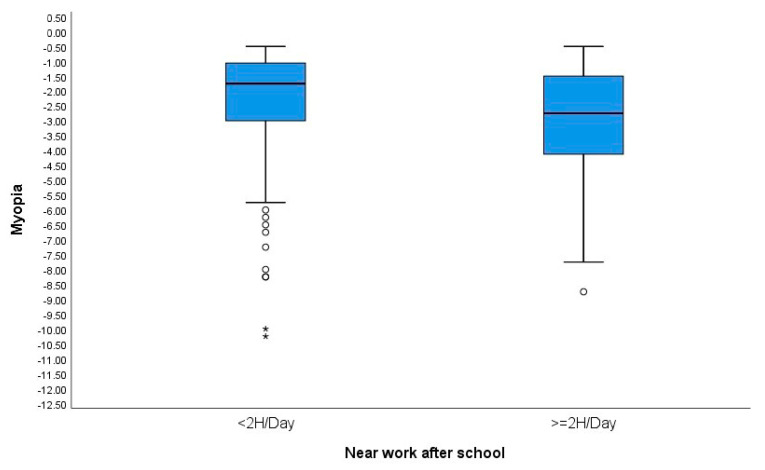
Boxplots showing the relationship between near work after school and the magnitude of refractive error (SER). °: outliers (values more than 1.5 box lengths away from the 75th percentile, or less than the 25th percentile) *: extreme cases (values more than 3 box lengths away from the 75th percentile, or less than the 25th percentile).

**Figure 2:**
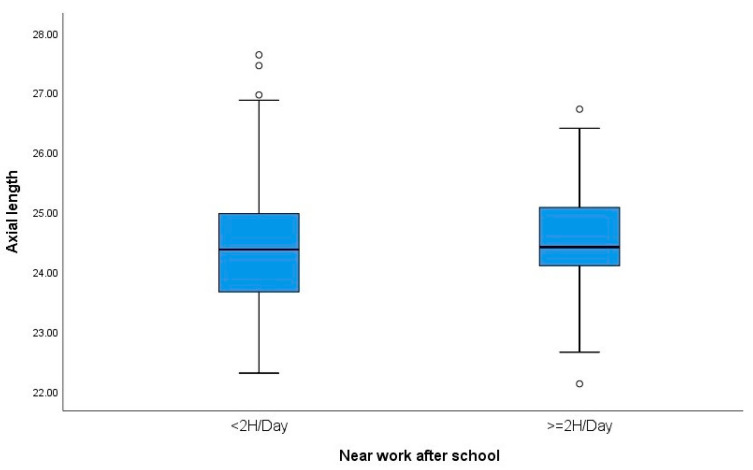
Boxplots showing the relationship between near work after school and axial length (AL). °: outliers (values more than 1.5 box lengths away from the 75th percentile, or less than the 25th percentile).

Regarding near work after school, participants were divided into two groups: those who spent less than 2 h, and those who spent more than 2 h per day. A statistically significant relationship was found between SER and near work. Subjects who spent less than 2 h on near work were less myopic (SE: −1.75 ± 1.83) than those who spent more than 2 h (−2.75 ± 1.82 D), *p* = 0.03. However, there were no differences in relation to axial length. Figure 1 and Figure 2 show boxplots of the relationship between near work performed after school and the magnitude of subjective refraction and axial length, respectively.

**Figure 3:**
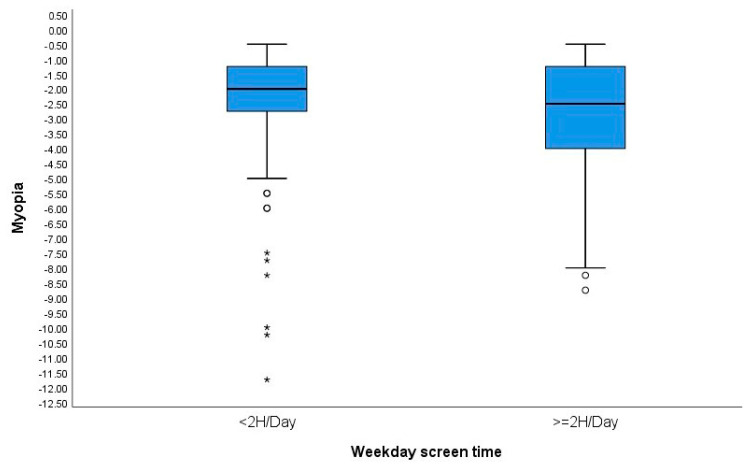
Boxplots showing the relationship between weekday screen time and the magnitude of refractive error (SER). °: outliers (values more than 1.5 box lengths away from the 75th percentile, or less than the 25th percentile). *: extreme cases (values more than 3 box lengths away from the 75th percentile, or less than the 25th percentile).

**Figure 4:**
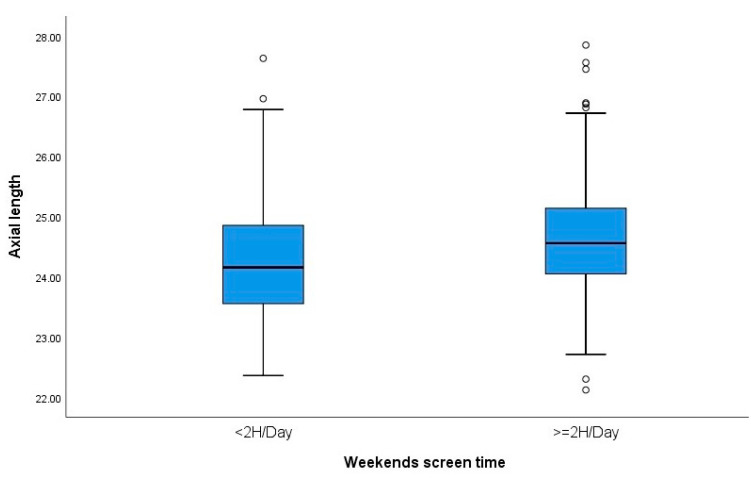
Boxplots showing the relationship between weekend screen time and axial length. °: outliers (values more than 1.5 box lengths away from the 75th percentile, or less than the 25th percentile).

Weekday and weekend screen time were evaluated. For this purpose, two groups were established: screen time <2 h/day and screen time more than 2 h/day. We observed that SE was significantly higher in subjects who spent more than 2 h per weekday on screen time compared to the group that spent less than 2 h/day (SER: −2.50 ± 1.88 D, −1.75 ± 1.91 D, respectively; *p* = 0.009). Subjects who had more than 2 h per day of screen time on weekends had longer AL than those who had less than 2 h (AL: 24.61 ± 0.96 mm, 24.33 ± 1.05 mm, respectively; *p* = 0.010). (Table 3) Figure 3 and Figure 4 show boxplots of the relationships between weekday screen time and the magnitude of subjective refraction and between weekend screen time and axial length, respectively.

Time spent outdoors during weekdays and weekends was divided into four groups: <1 h, 1–2 h, 2–4 h, >4 h. Most children spent less than 2 h a day outdoors (94% on weekdays and 69.4% at the weekend). Only 6% of children spent more than 2 h outdoors on weekdays, and 30.6% did so on weekends. No statistically significant results were obtained between refraction and axial length and hours spent outdoors (*p* > 0.05).

Another item of concern was the lighting the subjects had during near work at home: natural light, ceiling light, and desk lamp. A total of 80.8% of them had natural light, 84.2% of the children had ceiling lighting, and only 39.5% of all participants used desk lamps. Significant differences in SER and AL were only found in children who used desk lamps. Those who used desk lamps were more myopic and had greater AL (*p* = 0.025 and *p* = 0.02, respectively; Table 3).

## 4. Discussion

### 4.1. Clinical Data

Correlation of axial length with cycloplegic autorefraction

In this study, axial length was measured using biometry because it is a gold-standard measurement method. Axial length cannot be calculated from other optometric values, as erroneously suggested by some groups [26]. Our results showed that there was a moderate inverse correlation in both eyes between both variables, indicating that axial length increased moderately with myopia.

These results had also been obtained previously in CLEERE [27], a study in which 605 children 6 to 14 years of age who became myopic and 374 emmetropic children were evaluated, and the results obtained were that the children who became myopic had greater axial lengths than subjects in the emmetropic group before and after myopia onset, *p* < 0.0001. Similarly, in the Sydney Myopia Study [28], 12-year-old children underwent an ophthalmologic examination with ocular biometry measurements with IOL Master, and it turned out that the axial length of myopic eyes was generally greater than that of non-myopic eyes, *p* < 0.0001. In another large study in Singapore (SCORM), data were collected from 1775 Asian children aged 6 to 10 years in three visits and ocular biometry was compared in five established groups. It was found that axial length in children who had just developed myopia or had persistent myopia showed a faster elongation than in emmetropic children, *p* < 0.01 [29].

Therefore, following this research in the available literature, it might be assumed that an increase in axial length is related to myopia in children and, consequently, an increase in myopia leads to an increase in axial length.

Correlation of age with cycloplegic autorefraction

The analysis performed to evaluate the correlation between the age of the enrolled subjects and myopia showed that there was no correlation between these two variables. According to these results, this might indicate that age and refractive error are independent variables. However, previous studies have found that myopia rates increase with age [30,31,32]. The consistency of the aforementioned studies leads us to believe that the differences were due to the limited sample size. In addition, we had wide dispersion in the age groups, which prevented us from making larger subgroups.

### 4.2. Demographic and Lifestyle Factors

In recent years, different intervention methods have been investigated to control myopia progression in children. However, lifestyle-related risk factors associated with myopia should not be overlooked, as evidence from the past few decades suggests that environmental factors are driving the increase in myopia prevalence [12,33].

For this purpose, each of the lifestyle factors analyzed will be elaborated in the discussion section.

Parental myopia

In our study, the incidence of myopia in children was assessed according to whether they had a myopic parent, whether both parents were myopic, or whether neither parent was myopic, finding that 40.3% of the participants had no history of parental myopia. In addition, no significant association was found between parental myopia and both refractive error and axial length.

In contrast to our results, other authors point out that parental myopia is a key factor in the development of myopia. Jones. L. et al. evaluated 514 myopic children to predict future myopia in children based on parental myopia and concluded that almost half of future myopic children had both myopic parents (*p* < 0.001) and the risk of myopia increased depending on whether only one or both parents were myopic [34]. Likewise, the COMET research group and other studies confirmed the results obtained in the Orinda Longitudinal Study; children with myopia were more likely to have parents with myopia [35,36]. It has also been reported that the degree of parental myopia is the main independent influence on childhood myopia [5]. As a result, based on parental myopia data, children who are at high risk of myopia could be identified for early prevention strategies. The differences that we found with respect to our study may be associated with sample size limitations or the use of different criteria for measurement.

Age of onset of myopia and previous myopia control treatment

Considering the age of onset of myopia, our results show that children who began to be myopic before the age of 10 had more myopia than children whose age of onset was after 10 years. This is consistent with previous studies that have established that the age of onset of myopia is an important predictor of the final degree of myopia [37]. The CLEERE study provided data on the progression of 594 children, showing that younger children have faster myopia progression. For instance, their model predicted a 3-year progression of −1.93 D in an Asian American child when myopia started at the age of 7 years, as opposed to around −1.43 D when onset occurred at age of 10 years [31]. Bullimore et al. analyzed the results of several studies with Asian and non-Asian populations, showing that for European children who develop myopia at the age of 8, the mean myopia magnitude at 17 years old is expected to be approximately −4.00 D. Among seven non-East Asian studies, each later year of onset of myopia was associated with 0.23–0.50 D less myopia at the final recorded refraction [38]. The age of onset significantly influences the extent of myopia, with earlier onset allowing for a longer progression window. Therefore, efforts should focus on delaying onset and slowing progression, especially in younger children.

There were also significant differences between children who had followed previous myopia control treatment and those who had not, with the former being more myopic and with greater AL than those who had not previously been treated. These results may be explained by the greater concern of parents with more myopic children and their interest in reducing their progression.

School performance

Several environmental factors have been associated with myopia progression, including education, near work, season of birth, urbanization, socioeconomic status, and time spent outdoors. Among these, the two factors that have been demonstrated to play an important role in the progression of myopia are education and near work [33].

A substantial amount of data consistently support a causal relationship between educational level and myopia. A higher prevalence of myopia is associated with more years in education. Each additional year in education increases myopic refractive error by −0.27 D/year [39]. The European Eye Epidemiology Consortium [9] investigated the association between education and myopia in 61,946 patients from 15 studies, showing that educational level was significantly associated with myopia prevalence across all age strata. Education was significantly associated with myopia; the age-standardized prevalence was 25.4% for those who completed primary education, 29.1% for those who completed secondary education, and 36.6% for those with higher education. In this sense, school performance was also included in the questionnaire as one of the variables of interest and was classified into three groups: outstanding, adequate, and, finally, mild or severe difficulty. After statistical analysis, it was established that there were no significant differences between the three groups. Upon reviewing other studies, it was inferred that this is a very complex relationship to analyze, due to the different techniques used to measure school performance, since it is not a very reliable variable. In the future, it would be interesting to carry out more studies on this topic with a larger sample, previously establishing some criteria for its measurement and then being able to compare it with other existing studies. However, it is known that there is a trend of increasing prevalence of myopia as higher levels of education are considered, suggesting a potential effect of years of education. It is to be expected that a large part of the children of the average age in our study, 10 years old, will continue studying for many years. Furthermore, some confounding factors may be involved in the association between education and myopia, such as increased near-work activity and consequently less time spent outdoors. Thus, more data would need to be collected to obtain conclusive results.

Near work

The increased amount of near work as part of education promotes myopia progression. The current evidence supports that the duration and nature of near work as well as visual attitudes (working distance, duration) while performing near work influence myopia progression [12]. Dutheil F. et al. assessed the effects of near work on myopia by conducting a systematic review and meta-analysis, showing that the odds of myopia in the population exposed to near work is increased by 31% in children and by 21% in adults. Interestingly, they also found that there are no significant differences between paper reading and computer work in terms of myopia [40]. In multiple logistic regression analysis, Philipp D. et al. showed that myopia was significantly associated with longer near-work sessions after adjustment for age, sex, and socioeconomic status in German children and adolescents [41]. In this sense, our results agree, showing that children who spent more than 2 h on near work were more myopic than those who spent less than 2 h a day (*p* = 0.03). Although the mechanisms by which near work dictates myopia progression are not clearly understood, research suggest that a reduction in long-duration near-work activities could protect against increased myopia [12].

Screen time

In recent years, we have spent a significant proportion of our work and leisure time using screens. Electronic devices for gaming, digital entertainment, and social media have infiltrated deeply into our daily lives [42]. The average daily screen time for children aged 8 to 12 has seen a 49 min increase over the past three years. In 2016, the average daily screen time was 4 h and 18 min, and it rose to 5 h and 7 min in 2019 [43]. The prolonged use of electronic devices or screen time (time spent on computers, smartphones, tablets, etc.) has been proposed as a factor related to myopia development among children [42,44]. Since educational and work patterns vary between weekdays and weekends, we analyzed these data individually. Regarding weekday and weekend screen time, our findings show that myopia was significantly higher in subjects who spent more than 2 h per weekday using screens and AL was also significantly higher in those who had more than 2 h of screen time during the weekend compared to the group that had less than 2 h/day. Similarly, the group of CLEERE researchers also related time spent with electronic devices with an increase in myopia [31]; Enthoven et al. showed that episodes of 20 min of continuous use of smartphones were associated with more myopic refractive errors in Dutch teenagers [45]; and Alvarez-Peregrina et al. showed that Spanish myopic children aged between 5 and 7 have more screen time when compared to those without myopia [46]. This differs from the results of the Consortium [47], in which screen time was not significantly associated with myopia. But these results could be different if TV watching had been excluded from the analysis of their sample of almost 10.000 children who were assessed for screen time. It is known that TV watching is performed at a greater viewing distance compared to a computer screen, tablet, or mobile phone, and as was explained previously, myopia is related to near-work activities; watching TV is a distance activity and is not considered a risk factor for myopia [48].

Outdoor time

The best-established approach for delaying myopia onset is increasing the time spent outdoors. Epidemiological studies of large cohorts of children have shown a compelling relationship between more time outdoors and a lower incidence of myopia [34,49]. Likewise, randomized clinical trials have shown that encouraging outdoor activity during class recess can halve the incidence of myopia [50]. Most authors agree that the risk of onset of myopia could be decreased by increasing the time spent outdoors. Children with limited sunlight exposure face a 2.6 times higher risk of developing myopia [49]. While the exact mechanism of action is not fully understood, various theories propose that exposure to UV light triggers the release of dopamine, potentially leading to a reduction in axial length growth of the eye [12,17,33] But, paradoxically, outdoor time has not been shown to be effective in slowing progression in eyes that are already myopic [12,15,16,17,30,51,52]. Our results show a lack of correlation between outdoor time during weekdays and weekends and SER and AL. In the present study, there was no non-myopic comparative group, which could partially explain the lack of significance in the relationship between outdoor time during weekdays and weekends and SER and AL. Another potential factor in our findings is that 94% of the myopic children included in the present study spent ≤2 h a day outdoors during weekdays, and 69.4% spent ≤2 h a day outdoors during the weekend. Therefore, the time spent outdoors could be below the protective effect threshold of outdoor exposure.

Ref. [14]. In our study, a very small percentage of children spent more than 2 h outdoors on both weekdays (6%) and weekends (30.6%). These data are comparable with those found in a study with Spanish children which showed few children spent more than 2.5 h per day exposed to sunlight [21]. However, our study does not show a statistically significant relationship between outdoor time and SER or AL, whereas Alvarez Peregrina’s study found more time spent outdoors was associated with lower rates of myopia. The differences with respect to our study may be also associated with two factors: age of the participants and the questionnaire used. They included children younger than ours (between 5 and 7 years), and their results showed that the number of hours that children spend outdoors decreases with age; they may have obtained different results regarding outdoor time in older children. Related to the questionnaire, they categorized time spend outdoors as follows: low (0 to 1.6 h per day), moderate (1.6 to 2.7 h per day), or high (more than 2.7 h per day), which is a different segmentation of hours in the questionnaire. Possibly, a questionnaire with a more rigorous time limit would have been necessary and would have yielded more concrete and faithful results.

Lighting (related to near-work activities)

Cues derived from daily visual scenes, encompassing factors such as lighting and optical defocus across the visual stimuli field, have been proposed to influence the emmetropization process and, in turn, myopia onset and progression [53]. A widely accepted theory is related to light intensity. Animal studies suggest that higher light intensity can retard form deprivation and lens-induced myopia [54]. Wen J et al. carried out a clinical trial with Asian children showing that elevated light intensity in classrooms was related to low myopia incidence and progression over one year [17]

In this sense, we would have expected to find lower rates of myopia in those children who used a desk lamp in addition to a ceiling light, but our results show that those children who used a desk lamp were more myopic and had greater AL than those who did not use a desk lamp. Multiple factors could have influenced the results, such as the light intensity used in the desk lamp or the presence or absence of natural light in the room, which would require the use of an auxiliary light, such as a desk lamp. On the other hand, perhaps the children in our study who used desk lamps were the ones who spent more time on near work, and as previously discussed, near work is related to myopia.

Limitations

One of the limitations of this work is the number of patients included. In this sense, in the Myopia Unit of the HCSC, data on the lifestyle of myopic patients continue to be collected, so the sample is expected to expand in the future. On the other hand, this is a cross-sectional study, so there are no prospective data on these patients. In the long term, more results will be obtained to analyze these variables when the recruitment period and subsequent reviews end.

The most common source of risk of bias in studies that evaluate the relationship between screen time, near-work or outdoor activities, and myopia incidence or prevalence is the failure to include reliable measures for these variables. A recent systematic review and meta-analysis that included 33 studies on the relationship between screen use and the incidence and prevalence of myopia indicated that screen exposure was measured by use of questionnaires in all studies, with one also measuring device-recorded network data consumption [55]. Additional research utilizing objective measurements of screen time, near work, and outdoor activities is essential to evaluate the evidence supporting a potential connection between these variables and myopia. As technology advances, the assessment of near work should include more advanced methodology, going beyond the basic recording of distance, duration, or types of near tasks.

The segmentation by hours of our questionnaire encompasses a wide margin. Possibly, a questionnaire with a narrower time limit and one in which the hours of device use, hours outdoors, and school performance had been recorded more accurately would have yielded more specific and more accurate results. Perhaps other aspects should have been included, such as hours of sleep and physical activity, which may be of interest to include in the questionnaire in the future.

In cases where it is not possible to take objective measurements, it would be advisable to be able to unify the measurements with validated questionnaires translated into the language of the study population. Moreover, imposing more rigorous time limits would be necessary, resulting in more concrete and reliable outcomes.

## 5. Conclusions

Four main findings have been identified in this study. The first is that early age of onset of myopia is related to greater myopia. Second, greater screen time (more than 2 h on weekdays and weekend) was associated with higher rates of myopia and axial length. Third, a longer period of near work (more than 2 h a day after school) is associated with higher rates of myopia. Fourth, there was no relationship between outdoor time and the amount of myopia or axial length.

Our findings suggest that an earlier age of onset of myopia and prolonged screen and near-work activity time are related to higher rates of myopia, so reducing near work and screen time could serve as a valuable intervention that encourages healthier habits. Given that our results have not shown an association between outdoor time and the amount of myopia or axial length of the participants, it would be interesting to confirm these results through future research.

## Figures and Tables

**Table 1:** Personal and family history.

	N (%)
**Prematurity**	24 (7.5)
**Growth retardation**	22 (6.9)
**Current medical treatment**	58 (18.1)
**Ophthalmologic history**	21 (6.6)
**Previous ocular surgery**	4 (1.2)
**Parental myopia**	
None	128 (40.3)
Father or mother	115 (36.2)
Both	75 (23.6)
**Previous myopia control treatment**	75 (23.7)
**Iris color**	
Blue	9 (2.8)
Green	23 (7.2)
Light brown	41 (12.8)
Dark brown	247 (77.2)

**Table 2:** Lifestyle factors related to myopia.

	N (%)
**School performance**	
Outstanding	123 (39.3)
Adequate	149 (47.6)
Mild and severe difficulty	39 (12.5)
**School schedule**	
Split schedule	136 (51.3)
Intensive schedule	122 (46)
Mixed schedule	6 (2.3)
**Extracurricular activities**	
None	69 (26.1)
Outdoors	54 (20.5)
Indoors	102 (38.6)
Both	39 (14.8)
**Use of screens at school**	138 (43.7)
**Near-work time**	
**(after school)**	
<2 h	176 (67.4)
>2 h	85 (32.6)
**Screen time (weekdays)**	
<2 h	143 (45.1)
>2 h	174 (54.9)
**Screen time (weekends)**	
<2 h	76 (24.1)
>2 h	240 (75.9)
**Time spent outdoors (weekdays)**	
<1 h	217 (68.2)
1–2 h	82 (25.8)
2–4 h	18 (5.7)
>4 h	1 (0.3)
**Time spent outdoors (weekends)**	
<1 h	89 (28.1)
1–2 h	131 (41.3)
2–4 h	89 (28.1)
>4 h	8 (2.5)
**Access to green spaces out of school**	166 (63.6)
**Home lighting conditions**	
Natural light conditions	256 (80.8)
Ceiling illumination	267 (84.2)
Desk lamp	124 (39.5)

**Table 3:** Relationship between lifestyle factors and the magnitude of refractive error (SER) and axial length (AL).

	SER (D)	*p*-Value	AL (mm)	*p*-Value
**Medical background**				
No	−2.11 ± 1.90	0.161	24.54 ± 0.97	0.341
Yes	−2.50 ± 1.90		24.65 ± 0.82	
**Prematurity**				
No	−2.25 ± 1.90	NA	24.54 ± 0.96	NA
Yes	−2.50 ± 1.97		24.50 ± 1.28	
**Growth retardation**				
No	−2.13 ± 1.85	NA	24.54 ± 0.97	NA
Yes	−2.50 ± 2.28		24.44 ± 1	
**Parental myopia**				
None	−2.50 ± 1.82		24.61 ± 1.03	0.667 ^a^
Father or mother	−2.00 ± 1.91	0.057	24.56 ± 1.03	0.075 ^b^
Both	−2.00 ± 2.00		24.36 ± 0.78	
**Myopia onset age**				
<10 years old	−2.50 ± 1.99	*p* < 0.01	24.56± 0.96	0.873
>10 years old	−1.75 ± 1.44		24.54 ± 1	
**Previous ocular surgery**				
No	−2.25 ± 1.89	NA	24.53 ± 0.98	NA
Yes	−2.62 ± 3.07		24.94 ± 1.21	
**Current medical treatment**				
No	−2.25 ± 1.82	0.707	24.51 ± 1.02	0.341
Yes	−1.87 ± 2.25		24.65 ± 0.82	
**Ophthalmological history**				
No	−2.25 ± 1.92	NA	24.54 ± 0.98	NA
Yes	−2.00 ± 1.78		24.53 ± 1.13	
**Previous myopia control treatment**				
No	−1.75 ± 1.62	*p* < 0.001	24.43 ± 0.96	0.002
Yes	−3.00 ± 2.33		24.84 ± 1.01	
**School performance**				
Outstanding	−2.25 ± 2.00		24.50 ± 0.98	0.798 ^c^
Adequate	−2.25 ± 1.84	0.944	24.53 ± 0.96	0.511 ^d^
Mild and severe difficulty	−2.25 ± 1.75		24.62 ± 0.95	
**School schedule**				
Split schedule	−2.00 ± 1.73		24.42 ± 0.84	
Intensive schedule	−2.00 ±1.95	0.537	24.5 ± 1.05	0.472
Mixed schedule	−3.12 ± 1.85		24.92 ± 0.69	
**Use of screens at school**				
No	−2.00 ± 1.77	0.071	24.46 ± 1.01	0.135
Yes	−2.50 ± 2.05		24.63 ± 0.96	
**Extracurricular activities**				
None	−2.50 ±1.84	0.590	24.42 ± 0.98	0.552
Outdoors	−2.00 ± 2.20		24.66 ± 0.9	
Indoors	−2.00 ± 1.73		24.41 ± 0.93	
Both	−1.75 ± 1.49		24.43 ± 0.97	
**Near-work time (after school)**				
<2 h	−1.75 ± 1.83	0.03	24.45 ± 0.98	0.551
>2 h	−2.75 ± 1.82		24.52 ± 0.86	
**Screen time (weekdays)**				
<2 h	−2.00 ± 1.91	0.009	24.45 ± 1	0.172
>2 h	−2.50 ± 1.88		24.6 ± 0.97	
**Screen time (weekends)**				
<2 h	−2.00 ± 2.30	0.218	24.33 ± 1.03	0.010
>2 h	−2.25 ± 1.77		24.61 ± 0.96	
**Time spent outdoors (weekday)**				
<1 h 1	−2.25 ± 1.71	0.994	24.51 ± 0.96	0.609
1–2 h	−2.00 ± 2.15		24.52 ± 1.08	
2–4 h	−2.25 ± 2.72		24.77 ± 0.86	
>4 h	−4.50		25.11	
**Time spent outdoors (weekends)**				
<1 h	2.25 ± 1.65	0.769	24.52 ± 1.06	0.297
1–2 h	−2.00 ± 2.05		24.6 ± 0.96	
2–4 h	−2.00 ± 1.85		24.44 ± 0.94	
>4 h	−4.00 ± 1.68		24.44 ± 0.88	
**Natural light conditions**				
No	−2.25 ± 1.67	0.293	24.48 ± 1	0.622
Yes	−2.00 ± 1.96		24.55 ± 0.98	
**Ceiling illumination**				
No	−2.31 ± 2.01	0.548	24.47 ± 1.03	0.613
Yes	−2.25 ± 1.88		24.55 ± 0.98	
**Desk lamp**				
No	−2.00 ± 1.95	0.025	24.44 ± 0.99	0.02
Yes	−2.37 ± 1.82		24.68 ± 0.97	
**Access to green spaces outside school**				
No	−2.25 ± 2.06	0.462	24.5 ± 0.96	0.751
Yes	−2.00 ± 1.70		24.47 ± 0.92	

NA: *p*-value not applicable; (^a^): Statistically significant values between “None” and “Father or mother” (parental myopia); (^b^): Statistically significant values between “None” and “Both” (parental myopia); (^c^): Statistically significant values between “Outstanding” and “Adequate” (school performance); (^d^): Statistically significant values between “Outstanding” and “Mild and severe difficulty” (school performance).

## Data Availability

Data supporting the reported results can be acquired on request from the principal investigator.
